# Osteopontin Deletion Prevents the Development of Obesity and Hepatic Steatosis via Impaired Adipose Tissue Matrix Remodeling and Reduced Inflammation and Fibrosis in Adipose Tissue and Liver in Mice

**DOI:** 10.1371/journal.pone.0098398

**Published:** 2014-05-28

**Authors:** Andoni Lancha, Amaia Rodríguez, Victoria Catalán, Sara Becerril, Neira Sáinz, Beatriz Ramírez, María A. Burrell, Javier Salvador, Gema Frühbeck, Javier Gómez-Ambrosi

**Affiliations:** 1 Metabolic Research Laboratory, Clínica Universidad de Navarra, Pamplona, Spain; 2 CIBER Fisiopatología de la Obesidad y Nutrición (CIBERobn), Instituto de Salud Carlos III, Madrid, Spain; 3 Department of Histology and Pathology, University of Navarra, Pamplona, Spain; 4 Department of Endocrinology & Nutrition, Clínica Universidad de Navarra, Pamplona, Spain; Complexo Hospitalario Universitario de Santiago, Spain

## Abstract

Osteopontin (OPN) is a multifunctional extracellular matrix (ECM) protein involved in multiple physiological processes. OPN expression is dramatically increased in visceral adipose tissue in obesity and the lack of OPN protects against the development of insulin resistance and inflammation in mice. We sought to unravel the potential mechanisms involved in the beneficial effects of the absence of OPN. We analyzed the effect of the lack of OPN in the development of obesity and hepatic steatosis induced by a high-fat diet (HFD) using OPN-KO mice. OPN expression was upregulated in epididymal white adipose tissue (EWAT) and liver in wild type (WT) mice with HFD. OPN-KO mice had higher insulin sensitivity, lower body weight and fat mass with reduced adipose tissue ECM remodeling and reduced adipocyte size than WT mice under a HFD. Reduced MMP2 and MMP9 activity was involved in the decreased ECM remodeling. Crown-like structure number in EWAT as well as F4/80-positive cells and *Emr1* expression in EWAT and liver increased with HFD, while OPN-deficiency blunted the increase. Moreover, our data show for the first time that OPN-KO under a HFD mice display reduced fibrosis in adipose tissue and liver, as well as reduced oxidative stress in adipose tissue. Gene expression of collagens *Col1a1*, *Col6a1* and *Col6a3* in EWAT and liver, as well as the profibrotic cytokine *Tgfb1* in EWAT were increased with HFD, while OPN-deficiency prevented this increase. OPN deficiency prevented hepatic steatosis via reduction in the expression of molecules involved in the onset of fat accumulation such as *Pparg*, *Srebf1*, *Fasn*, *Mogat1*, *Dgat2* and *Cidec*. Furthermore, OPN-KO mice exhibited higher body temperature and improved BAT function. The present data reveal novel mechanisms of OPN in the development of obesity, pointing out the inhibition of OPN as a promising target for the treatment of obesity and fatty liver.

## Introduction

Changes in lifestyle and diet have caused over the last decades a progressive increase in the incidence of obesity, being one of the most prevalent metabolic disorders. Obesity is associated with increased morbi-mortality from conditions such as type 2 diabetes, cardiovascular disease, hyperlipidemia, steatohepatitis and cancer [1].

Osteopontin (OPN, *Spp1*), is a multifunctional extracellular matrix-associated protein abundantly expressed in bone, being also expressed in other cell types such as macrophages, smooth muscle cells and hepatocytes [2]. OPN expression is upregulated by proinflammatory cytokines such as tumor necrosis factor-α (TNF-α) and transforming growth factor-β (TGF-β), as well as by hypoxia and hyperglycemia [2]. OPN binds to integrin receptors and CD44 mediating cell-matrix and cell-cell interactions [3]. Besides its function as a key molecule regulating bone mineralization [4], OPN is also involved in the immune and inflammatory responses, playing an active role in the development of cardiovascular disease, diabetes, fatty liver disease and cancer [2], [3], [5].

We have previously shown that OPN is produced by adipose tissue and that OPN expression is dramatically increased in visceral adipose tissue in obesity [6], [7]. Subsequently, others have confirmed our findings showing that OPN is heavily involved in the obesity-associated proinflammatory state and insulin resistance [8]–[14], although the mechanisms involved have not been fully elucidated. Thus, the aim of our study was to analyze the effect of the absence of OPN in the development of obesity induced by a high-fat diet (HFD) in mice to unravel the potential mechanisms involved. Herein we report that mice lacking OPN are protected against the development of diet-induced obesity through mechanisms involving impairment of adipose tissue extracellular matrix remodeling, reduction in fibrosis and inflammation in adipose tissue and liver, and improvement in brown adipose tissue (BAT) function.

## Materials and Methods

### Ethics Statement

This study was carried out in strict accordance with the European Guidelines for the Care and Use of Laboratory Animals and was approved by the Ethical Committee for Animal Experimentation of the University of Navarra (071/07).

### Animals and treatment

Ten-week old male wild type (C57BL/6J) (n = 18) and OPN-knockout [*Opn* ^-/-^(B6.Cg-Spp1^tm1blh^/J (The Jackson Laboratory)] (n = 18) were housed with controlled temperature (22±2°C), relative humidity (50%) and lighting (12∶12 h cycle of light-darkness, lights on at 08:00 am). Half of the animals were fed for 20 weeks with a commercial HFD [fat (60%), 23 kJ/g, Product # F3282, BioServe] and the other half with a chow diet [fat (13%), 12 kJ/g, 2014 Teklad diet, Harland Laboratories] [15]. The body weight of the animals and the amount of food eaten were registered every 3 days. Mice were sacrificed by CO_2_ inhalation after 6 h of fasting following the 20 week experimental period. After sacrifice, blood was obtained by cardiac puncture, body weight was recorded and white adipose tissue from different depots (epididymal, perirenal and subcutaneous) carefully dissected and weighed together with that of other organs. Serum and tissues were frozen at −80°C for subsequent experiments.

### Body temperature

Body temperature was determined at the end of the study by measuring the rectal temperature using a thermoprobe (YSI 4600 Thermometers, Yellow Springs Instruments).

### Intraperitoneal glucose tolerance tests and intraperitoneal insulin tolerance tests

The animals were fasted overnight prior to the tests. At 10:00 am glucose was measured at baseline in blood taken from the tail. Mice given 2 g of glucose/kg body weight (intraperitoneal glucose tolerance tests-IPGTT) or 75 of U insulin/kg body weight (intraperitoneal insulin tolerance tests-IPITT). Blood glucose was measured at 15, 30, 60 and 120 min.

### Blood analysis

Serum glucose concentrations were measured using a sensitive-automatic glucose sensor (Ascensia Elite, Bayer). Concentrations of triglycerides, total cholesterol (Infinity, Thermo Electron), free fatty acids (FFA) (WAKO Chemicals) and glycerol (Sigma) were measured by enzymatic methods using commercially available kits. Insulin and leptin were determined using mouse enzyme immunoassay ELISA kits (Crystal Chem) [16]. Insulin resistance was calculated using the HOMA index. Adiponectin (BioVendor), testosterone (R&D Systems), osteopontin (R&D Systems), resistin (Immuno-Biological Laboratories), corticosterone (Immuno-Biological Laboratories), ghrelin (Linco) and SAA (Biosource) concentrations were assessed using ELISA kits. Intra- and inter-assay coefficients of variation for measurements of the ELISA kits ranged between 2.6–4.2% for the former, and 5.3–8.1%, for the latter.

### Thiobarbituric acid reactive substances

Determination of lipid peroxidation was measured as previously described [17]. We used serum MDA levels as an indicator of lipid peroxidation and oxidative stress. Briefly, 5 µL of serum or standard (MDA) were mixed with 120 µL of diethyl thiobarbituric acid (DETBA) 10 mmol/L and then vortexed and incubated for 1 h at 95 °C. Vials were cooled 5 min at room temperature (RT) and 360 µL of *n*-butanol were added to DETBA-MDA adducts. Samples were shaken with vortex for 1 min and centrifuged for 10 min at 1,600 *g* at RT. Then, 250 µL of supernatant were read on 96-well plates on a Fluroskan Ascent (Thermo Labsystems) with 535 nm and 590 nm excitation and emission wavelength, respectively.

### RNA extraction and microarray experiments and analysis

RNA isolation from liver and adipose tissue was performed by homogenization with an ULTRA-TURRAX T 25 basic (IKA Werke GmbH) using respectively TRIzol (Invitrogen) and QIAzol Reagent (Qiagen). Samples were purified with the RNeasy Mini Kit and RNeasy Lipid Tissue Mini Kit (Qiagen) and treated with DNase I (RNase-free DNase Set, Qiagen) in order to remove any trace of genomic DNA. For first strand cDNA synthesis constant amounts of 2 µg of total RNA were reverse transcribed in a final volume of 40 µL using random hexamers (Roche) as primers and 400 units of M-MLV reverse transcriptase (Invitrogen) as previously described [18].

Gene expression profile analyses were performed using the Agilent Whole Mouse Genome array (G4121B, Agilent Technologies) as previously described [18], [19]. Five animals were used per group. Slides were scanned with a GenePix 4100A scanner (Axon Instruments) and images and data were analyzed using GenePiX Pro 6.0 and GeneSpring GX software v 7.3.1 (Agilent), respectively. Functional annotation networks were generated using the *Ingenuity Pathway Analysis* (IPA, Ingenuity Systems).

### Real-Time PCR

RNA was extracted as described above and transcript levels were quantified by Real-Time PCR (7300 Real Time PCR System, Applied Biosystem). Primers and probes (Table S1) were designed using the software Primer Express 2.0 (Applied Biosystems) and purchased from Genosys (Sigma). Primers or TaqMan probes covering fragments of the areas from the extremes of two exons were designed to ensure the detection of the corresponding transcript preventing genomic DNA amplification. The cDNA was amplified at the following conditions: 95°C for 10 min, followed by 45 cycles of 15 s at 95 °C and 1 min at 59 °C, using the TaqMan Universal PCR Master Mix (Applied Biosystems). The primer and probe concentrations for gene amplification were 300 and 200 nmol/L, respectively. The results were normalized to the levels of the *18S* rRNA (Applied Biosystems) and relative quantification was calculated using the ΔΔ*Ct* formula [6], [20]. Relative mRNA expression was expressed as fold expression over the calibrator sample (average of gene expression corresponding to the wild type group). All samples were run in triplicate and the average values were calculated.

### Western blot

Samples of epididymal white adipose tissue (EWAT) and liver were homogenized in RIPA buffer [1 mol/L Tris-HCl pH 7.40, 150 mmol/L NaCl, 1% Triton X-100, 0.1% sodium dodecyl sulphate (SDS), 5 mmol/L EDTA 2H_2_O, 1% deoxycolate] and supplemented with protein inhibitors (Complete™ Mini-EDTA free, Roche). The soluble proteins were extracted after centrifugation at 16,000 *g* for 15 min at 4 °C. The protein concentration was determined by the method of Bradford using bovine serum albumin (BSA) (Sigma) as standard. Equal amounts of protein (30 µg) were run out in 12% SDS-PAGE, subsequently transferred to nitrocellulose membranes (Bio-Rad Laboratories) and blocked in Tris-buffered saline (10 mmol/L Tris-HCl, 150 mmol/L NaCl, pH 8.00) with 0.05% Tween 20 (TBS-T) containing 5% non-fat dry milk for 1 h at RT. Blots were then incubated overnight at 4 °C with primary antibodies against AKT1-p (Ser473), AKT1 (Upstate), AMPK-p (Thr172), AMPK, ACC-p (Ser79), ACC, FAS, ATGL (Cell Signaling), HSL-p (Ser554), HSL, MMP2, MMP9, NOX2, ANXA2, UCP3, UCP1 (Abcam), UCP2 (Millipore) and AQP7 (Santa Cruz Biotechnology). Anti β-actin antibody (Sigma) was used for the normalization of density values. The antigen-antibody complexes were visualized using horseradish peroxidase-conjugated anti-goat (Zymed), anti-rabbit or anti-mouse IgG antibodies (Amersham Biosciences) and the enhanced chemiluminescence ECL detection system (Amersham biosciences). The intensity of the bands was determined by densitometric analysis with the Gel Doc™ gel documentation system and the Quantity One 4.5.0 software (Bio-Rad).

### Gelatin zymography

MMP2 and MMP9 gelatinolytic activities were measured as previously described [21]. Briefly, protein extracts of 15 µg from each sample were run in 10% SDS-PAGE containing 0.1% gelatin (Sigma). After the electrophoresis, gels were washed in 2.5% Triton X-100 (Sigma) for 45 min and subsequently incubated overnight at 37 °C in enzyme development buffer (Invitrogen). After incubation, gels were fixed in 50% (v/v), methanol and 7% (v/v) acetic acid (Sigma) for 15 min and then stained for 1 h in GelCode Blue Stain Reagent (Pierce). Finally, the gels were cleared in distilled water. Mmp-9 and Mmp-2 complex were identified based on their molecular weight and Quantity One (Bio-Rad) was used for densitometric analysis of the zymographic activities.

### Histological analysis

EWAT (6 µm), BAT (6 µm) or liver (4 µm) sections of tissue previously fixed in formalin and embedded in paraffin, were deparaffinized with xylene and hydrated with decreasing concentrations of ethanol. Samples were stained with hematoxylin-eosin or Sirius red. The sections were dehydrated with increasing concentrations of ethanol and xylene, mounted in DePex (Panreac) and observed with an optical microscope (Axiovert 40 CFL, Zeiss). The size of adipocytes and lipid droplets was determined by analyzing the cross-sectional area of white and brown adipose tissue with the software AxioVision 4.6 (Zeiss). Images of five fields per section from each animal were captured with a 200X magnification, and the adipocyte cell surface areas (H/E) from, at least, 100 cells/section or fibrotic streaks (Sirius red) were measured.

### Immunohistochemistry

Sections of formalin-fixed paraffin-embedded EWAT (6 µm) or liver (4 µm) were dewaxed with xylene and hydrated in decreasing concentrations of ethanol. Endogen peroxidase activity was quenched using 3% H_2_O_2_ (Sigma) in absolute methanol for 20 min at RT, and washed 3 times with ethanol. Sections were immersed in 10 mmol/L citrate buffer (pH 6.00) and heated using a microwave oven at 800 W for 10 min to enhance antigen retrieval. After cooling, sections were blocked for 1 h at RT in a humidified chamber with 5% goat serum (Sigma) in TBS. Sections were subsequently incubated with rat anti-mouse F4/80 antibody (AbD serotec) at a dilution of 1∶100 (EWAT) or 1∶50 (liver) in TBS with 2% goat serum (Sigma) in a humidified chamber overnight at 4 °C. After washing with TBS (3×5 min), sections were incubated with horseradish peroxidase-conjugated secondary anti-rat antibody (1∶200) (Amersham Biosciences) diluted in TBS with 2% goat serum for 1 h at RT. After washing with TBS (3×5 min), localization of the antigen-antibody complexes was performed by adding diaminobenzidine (DAB) (Amersham Biosciences). Negative control slides with omission of the primary antibodies were included in the immunostaining procedure. The reaction was stopped and contrasted with Harris hematoxylin solution (Sigma). Sections were dehydrated with increasing concentrations of ethanol and xylol, mounted in DePeX and observed with an optical microscope (Axiovert 40 CFL). The quantification of F4/80 positive cells in EWAT and liver, and crown-like structures (CLS) in EWAT content in 5 samples/group were analyzed using a double-blind protocol. The total number of F4/80 expressing cells and the total number of cells were counted in 5 slides (original magnification ×200 in EWAT and ×100 in liver) of each sample using the image analysis program AxioVision 4.6. The number of macrophages and total cells in each sample provided the percentage of F4/80 positive cells for each section analyzed.

### Intrahepatic lipid content

The hepatic triglyceride content was measured by enzymatic methods, in accordance with previously published procedures [22]. Briefly, tissues were homogenized and diluted in saline at a final concentration of 50 mg/mL. Homogenates were diluted (1∶1) in 1% deoxycholate (Sigma) and incubated at 37 °C for 5 min. For triglyceride measurements, samples were diluted 1∶100 in the reagent (Infinity Triglycerides Liquid Stable Reagent, Thermo Electron) and incubated for 30 min at 37 °C. The resulting dye was measured based on its absorbance at 550 nm with a Sunrise ELISA plate reader (Tecan). Concentrations were determined compared with a standard curve of triglycerides (Infinity Triglycerides Standard, Thermo Electron). The protein content of the preparations was measured by the Bradford method, using BSA (Sigma) as standard. All assays were performed in duplicate.

### Statistical analysis

Data are presented as mean ± SEM. The analysis of differences between experimental groups was performed by two-way ANOVA (genotype x diet) or by one-way ANOVA followed by Tukey HSD *post-hoc* tests, where appropriate. Statistical comparisons for microarray data to identify differentially expressed genes across different groups were performed using two-way ANOVA. The calculations were performed using the SPSS statistical package for Windows version 15.0.1 (SPSS). A *P* value less than 0.05 was considered statistically significant.

## Results

### OPN-deletion prevents HFD-induced increase in body weight and adipose tissue mass

**Figure 1:**
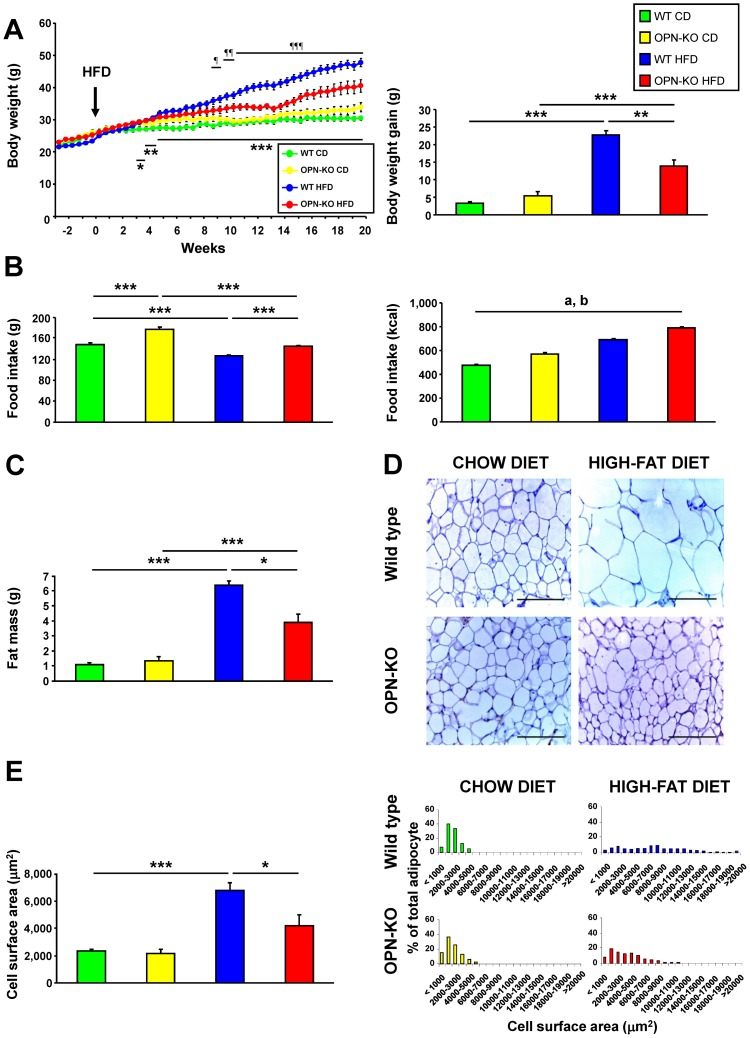
OPN-Deficiency prevents HFD-induced increase in body weight and adipose tissue mass. (A) Body weight evolution of the different experimental groups and weight gain of the animals from the different experimental groups after 20 weeks under CD or HFD. The arrow indicates the start of the HFD. *P<0.05, **P<0.01 and ***P<0.001 WT-CD *vs* WT-HFD. ¶*P*<0.05, ¶¶*P*<0.01 and ¶¶¶*P*<0.001 WT-HFD *vs* OPN-HFD. Mean ± SEM of 8–10 animals. (B) Cumulative food intake expressed as weight of food (g) or total energy (kcal) during the 20-week experimental period. Mean ± SEM of 8–10 animals. (C) Adipose mass (sum of epididymal, perirenal and subcutaneous depots) of the animals from the different experimental groups after 20 weeks under CD or HFD. Mean ± SEM of 8–10 animals. (D) Representative images of histological sections corresponding to EWAT from mice of different groups. The sections were stained with hematoxylin-eosin (H–E). Magnification 200X. Scale bar, 100 µm. (E) Cell surface area and distribution by areas of adipocytes in EWAT determined in histological sections of the different experimental groups after 20 weeks under the CD or HFD. Mean ± SEM of 5 animals. Statistical differences were determined by two-way ANOVA, ^a^ *P*<0.05, effect of OPN deficiency; ^b^ *P*<0.05 effect of diet. If an interaction was detected one-way ANOVA followed by Tukey's HSD test was performed, *P<0.05, **P<0.01 and ***P<0.001.

OPN-KO mice showed significant differences compared with WT mice in body weight since the ninth week under the HFD. Weight gain during the 20 weeks under the HFD was significantly lower in OPN-KO mice (Fig. 1A). OPN-deficiency influenced the weight of most of the studied organs (Table S2). Interestingly, OPN-KO mice exhibited a significantly higher food intake than WT mice reported either as weight of food eaten or amount of energy (Fig. 1B).

Serum and mRNA levels of OPN (*Spp1*) were, as expected, undetectable in KO animals. No differences in serum OPN concentrations in WT mice exposed to HFD were observed. However, mRNA expression of *Spp1* was significantly increased in EWAT and liver (30- and 1.7-fold, respectively) from WT mice exposed to HFD. Transcript levels of the OPN receptor *Cd44* increased after the HFD in adipose tissue and liver, but remained at normal levels in OPN-KO mice (Fig. S1).

Adipose mass (sum of epididymal, perirenal and subcutaneous depots) was significantly lower in OPN-KO mice than in WT mice with HFD (Fig. 1C). Furthermore, the EWAT adipocyte size was significantly lower in animals lacking OPN than in WT mice under HFD, which exhibited a lower percentage of large adipocytes than WT mice (Fig. 1D–E). Exposure to the HFD resulted in increased serum levels of leptin and corticosterone, which were significantly reduced in mice lacking OPN (Table 1). These results evidence that OPN is necessary for HFD-induced adipose tissue expansion.

**Table 1:** Metabolic Characteristics of Experimental Animals.

	Chow diet		High-fat diet	
	WT	OPN-KO	WT	OPN-KO
Glucose (mg/dL) ^b, c^	130±11	175±18	243±12^***,†^	215±17^**^
Insulin (ng/mL) ^a, b, c^	0.58±0.04	0.63±0.15	3.94±0.43^***,†††^	1.80±0.39^‡‡‡^
HOMA ^a, b, c^	4.4±0.4	7.2±2.2	58.5±8.9^***,†††^	25.5±7.0^‡‡^
Glycerol (mg/dL) ^b, c^	0.036±0.002	0.038±0.003	0.046±0.002^***^	0.038±0.002^‡^
FFA (mmol/L) ^a^	0.68±0.06	0.62±0.03	0.64±0.02	0.51±0.03
TG (mg/dL) ^b^	103±6	96±10	92±3	74±5
Cholesterol (mg/dL) ^a, b, c^	130±3	114±7	222±6^***,†††^	153±10^††,‡‡‡^
Leptin (ng/mL) ^a, b, c^	3.5±2.4	4.8±5.8	35.3±2.0^***,†††^	19.6±11.4^***,†††,‡‡‡^
Resistin (ng/mL) ^b^	14.2±1.9	12.5±1.3	18.1±2.9	22.3±2.9
Adiponectin (µg/mL) ^a^	22.0±1.4	17.8±1.1	27.4±1.4	17.6±1.6
Corticosterone (nmol/L) ^a, b^	381±31	304±63	562±38	347±52
Testosterone (ng/mL)	0.67±0.16	0.78±0.20	0.91±0.25	1.42±0.29
Total ghrelin (ng/mL) ^a, b^	1.60±0.41	2.42±0.44	0.75±0.08	1.31±0.19
SAA (µg/mL)	4.4±0.5	4.3±0.3	8.6±2.5	5.9±1.3

Mean ± SEM of 8–10 animals. Statistical differences were determined by two-way ANOVA. ^a^ *P*<0.05, main effect of OPN-deficiency; ^b^ *P*<0.05, main effect of diet; ^c^ *P*<0.05, interaction between factors. When interaction was detected, data were analyzed by one-way ANOVA followed by Tukey's HSD test. **P<0.01 and ***P<0.001 *vs* WT on CD; ^†^ *P*<0.05 and ^†††^ *P*<0.001 *vs* OPN-KO on a CD; ^‡^ *P*<0.05, ^‡‡^ *P*<0.01 and ^‡‡‡^ *P*<0.001 *vs* WT on HFD.

HFD and OPN-deficiency did not cause any disturbance in the amount of proteins involved in lipogenesis or lipolysis, nor in *Pparg* expression (Fig. S2A–G). We conclude that the changes observed in adipose mass are unlikely to be related with alterations in lipolysis or lipogenesis.

### Lack of OPN improves insulin sensitivity in mice fed with HFD

HFD resulted in increased serum levels of glucose, insulin and HOMA, which were significantly reduced in mice lacking OPN (Table 1). The IPGTT showed that mice under the HFD exhibited increased blood glucose levels, but no differences were detected by the lack of OPN. However, the IPITT showed that WT mice subjected to HFD had increased blood glucose levels while glucose concentrations of OPN-KO mice remained similar to the levels of WT mice (Fig. S3A–B).

**Figure 2:**
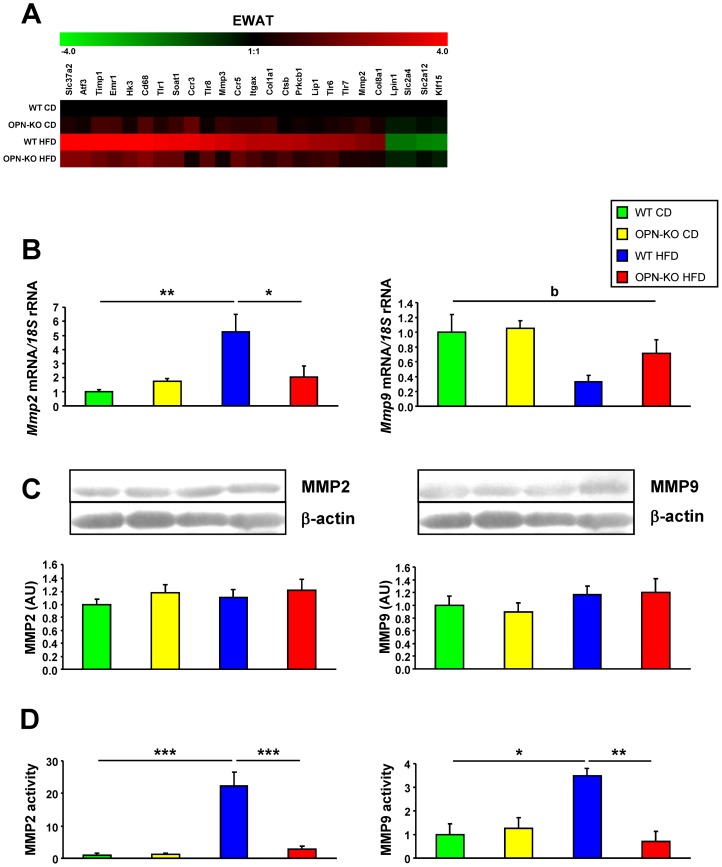
OPN-deficiency decreases MMP2 and MMP9 activity in adipose tissue. (A) Heat map showing changes in expression of selected genes in EWAT. Red and green colors represent up- and down-regulated expression, respectively on a log_2_ scale. (B) Gene expression levels of *Mmp2* and *Mmp9* in EWAT. (C) Protein expression levels of MMP2 and MMP9 in EWAT. (D) Zymography analysis of MMP2 and MMP9 activity in EWAT after 20 weeks of exposure to the chow diet or HFD. Mean ± SEM of 8–10 animals. Statistical differences were determined by two-way ANOVA, ^b^ *P*<0.05 effect of diet. If an interaction was detected one-way ANOVA followed by Tukey's HSD test was performed, *P<0.05, **P<0.01 and ***P<0.001.

Microarray gene expression profiling of EWAT, showed that OPN-deficiency prevented the HFD-induced decrease in mRNA levels of *Slc2a4* (GLUT4) and *Slc2a12* (GLUT12) (Fig. 2A and Table S3), which could be related to the improvement of glucose metabolism. To analyze the implication of skeletal muscle in the improvement of insulin sensitivity by the lack of OPN, gene expression levels of *Irs1*, *Irs2*, *Slc2a4* and *Ucp3* in gastrocnemius muscle were evaluated. *Slc2a4* levels decreased with HFD, but no other changes due to diet or the absence of OPN were observed (Fig. S3C–F).

### OPN-deletion decreases MMP2 and MMP9 activity in adipose tissue

Matrix metalloproteinases (MMPs) are extracellular proteolytic enzymes involved in adipose tissue expansion [21]. Functional annotation network from IPA revealed an important role of MMPs in the action of OPN in HFD-induced adipose tissue expansion (Fig. S4). In order to assess the involvement of MMPs in adipose tissue extracellular matrix remodeling, we studied gene and protein expression levels as well as activity of MMP2 and MMP9. *Mmp2* mRNA increased with HFD in the WT mice while OPN-deficiency prevented this increase (Fig. 2A–B). Protein expression of MMP2 and MMP9 was not affected either by HFD or OPN-deficiency (Fig. 2C). Interestingly, the gelatinase activity of MMP2 and MMP9 was dramatically increased with HFD, and this effect was prevented by OPN-deficiency (Fig. 2D). These data are consistent with a deficit in extracellular matrix remodeling in OPN-KO mice with HFD.

### Lack of OPN decreases inflammation, oxidative stress and fibrosis in adipose tissue

**Figure 3:**
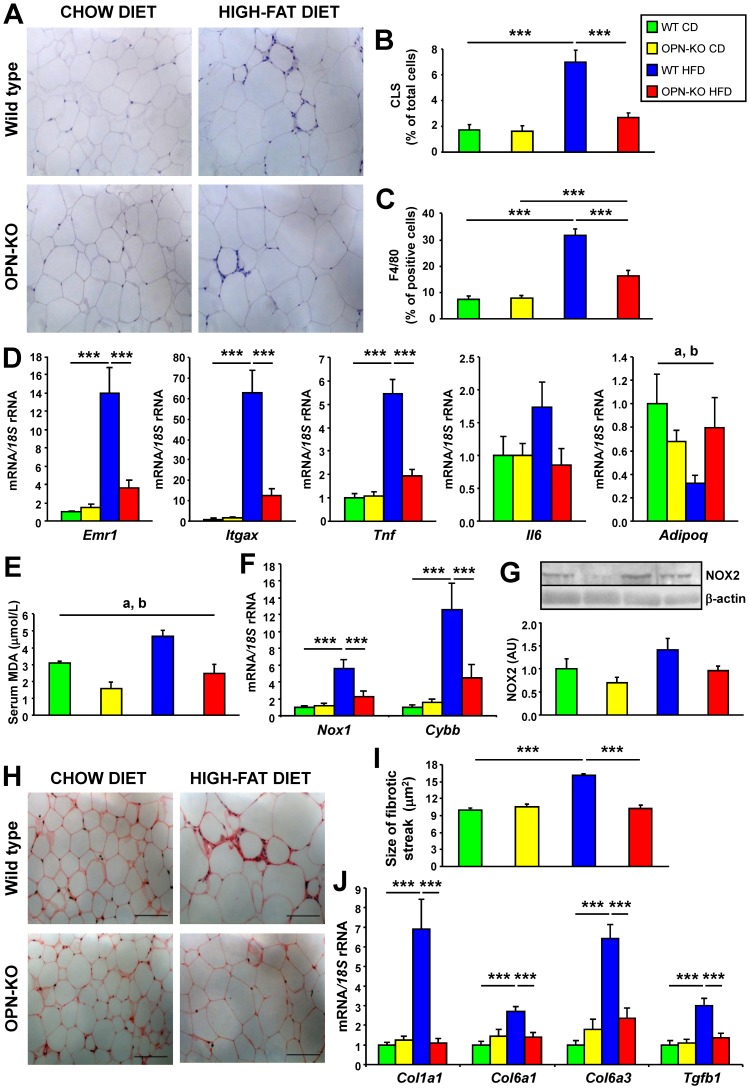
OPN-deficiency decreases inflammation, oxidative stress and fibrosis in adipose tissue. (A) Representative immunohistochemical staining of EWAT against the specific macrophage marker F4/80. Magnification 200X. Mean ± SEM of 5 animals. (B) CLS content determined by F4/80 positive staining and (C) percentage of F4/80 positive cells. Mean ± SEM of 5 animals. (D) Gene expression levels of *Emr1, Cd11c (Itgax), Tnf, Il6* and *Adipoq* in EWAT. Mean ± SEM of 8–10 animals. (E–G) Oxidative stress in serum and EWAT. TBARS in serum (E), *Nox1* and *Nox2* (*Cybb*) mRNA (F) and NOX2 protein (G) in EWAT after 20 weeks under the CD or HFD. (H–J) Fibrosis in EWAT. (H) Representative images of histological sections from EWAT stained with Sirius red. Magnification 200X. Scale bar, 100 µm. (I) Cell surface area of fibrotic streak in EWAT determined in histological sections. Mean ± SEM of 5 animals. (J) Expression of *Col1a1*, *Col6a1*, *Col6a3* and *Tgfb1* mRNA, genes involved in fibrosis, in EWAT. Mean ± SEM of 8–10 animals. Statistical differences were determined by two-way ANOVA, ^a^ *P*<0.05, effect of OPN deficiency; ^b^ *P*<0.05 effect of diet. If an interaction was detected one-way ANOVA followed by Tukey's HSD test was performed, ***P<0.001.

CLS number increased with HFD, while OPN-deficiency blunted the increase (Fig. 3A–B). The number of macrophages in EWAT, as evidenced by the higher number of F4/80-positive cells and *Emr1* expression, increased with HFD and OPN-deficiency partially prevented this increase (Fig. 3C–D). CD11c (*Itgax*) gene expression, a marker of M1 macrophage proinflammatory polarization [23], increased with HFD in EWAT, a phenomenon that was not observed with OPN-deficiency (Fig. 3D). Moreover, *Tnf* mRNA increased with HFD and OPN-deficiency prevented this increase. *Il6* mRNA and serum levels of the acute-phase reactant SAA showed the same trend, although the differences were not significant (Fig. 3D and Table 1). *Adipoq* mRNA decreased with HFD, and OPN-deficiency seemed to prevent this effect (Fig. 3D).

We next examined the levels of oxidative stress. HFD significantly increased serum TBARS concentrations, while OPN-deficiency prevented this increase (Fig. 3E). Mice under the HFD exhibited increased mRNA levels of *Nox1* and *Cybb* and NOX2 protein with OPN-deletion protecting against these increments (Fig. 3F and G). The decreased number and proinflammatory profile of macrophages, reduced expression of proinflammatory cytokines and NADPH components as well as lower lipid peroxidation indicate that OPN-deficiency protects against HFD-induced adipose tissue inflammation and oxidative stress.

Many studies have shown that obesity and diabetes are related to fibrosis in adipose tissue and liver [24], [25]. Whereas collagen fiber staining with Sirius red in adipose tissue obtained from WT mice with CD showed very thin collagen sheets surrounding adipocytes, adipose tissue from WT mice with HFD contained very pronounced fibrotic streaks among adipocytes. OPN-deficiency reduced the thickness of the fibrotic streaks (Fig. 3H–I). Gene expression of collagens *Col1a1*, *Col6a1* and *Col6a3* and profibrotic cytokine *Tgfb1* were increased with HFD, while OPN-deficiency prevented this increase (Fig. 2A and 3J and Fig. S4). The decrease of fibrotic streaks together with the decreased expression of collagens and markers of fibrosis indicate that OPN-deficiency protects against diet-induced fibrosis in adipose tissue.

### Lack of OPN prevents HFD-induced liver lipid accumulation

**Figure 4:**
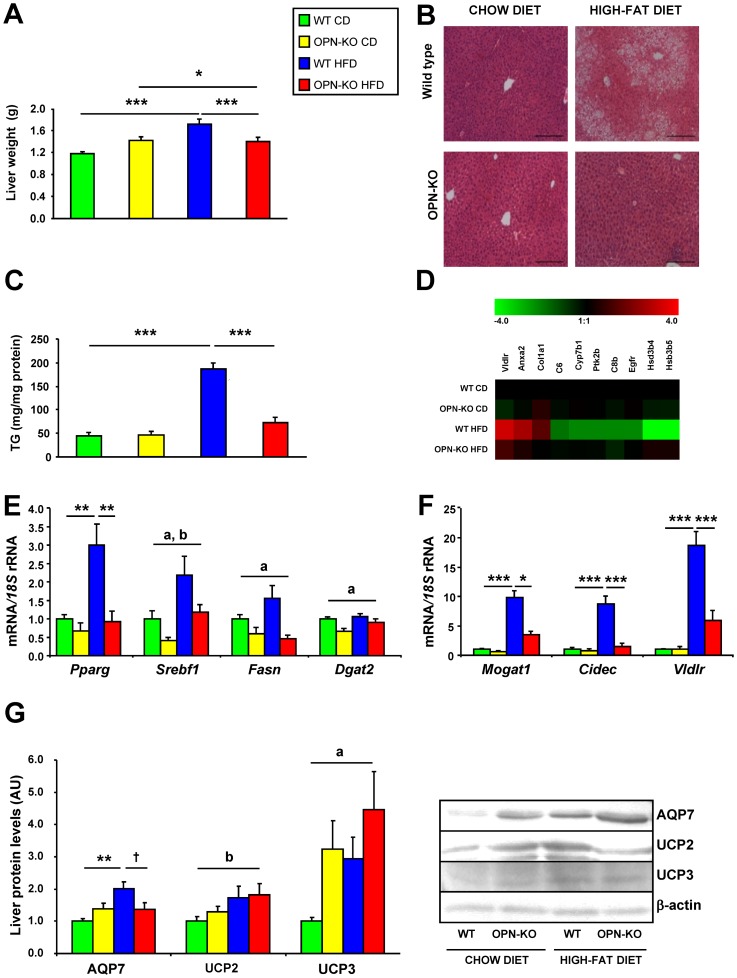
Lack of OPN prevents HFD-induced liver lipid accumulation. (A) Liver weight of the animals from the different experimental groups after 20 weeks of exposure to the chow diet or HFD. Mean ± SEM of 8–10 animals. (B) Representative images of histological sections from the liver of mice of different groups. The sections were stained with H–E. Magnification 100X. Scale bar, 200 µm. (C) Triglyceride content in the liver. (D) Heat map showing changes in expression of selected genes in liver. Red and green colors represent up- and down-regulated expression, respectively on a log_2_ scale. (E and F) Expression of lipogenic genes in the liver. *Pparg*, *Srebf1*, *Fasn*, *Dgat2* (E), *Mogat1, Cidec* and *Vldlr* (F). (G) Levels of proteins involved in liver steatosis. AQP7, UCP2, and UCP3 in liver after 20 weeks of exposure to the chow diet or HFD. Mean ± SEM of 8–10 animals. Statistical differences were determined by two-way ANOVA, ^a^ *P*<0.05, effect of OPN deficiency; ^b^ *P*<0.05 effect of diet. If an interaction was detected one-way ANOVA followed by Tukey's HSD test was performed, ^†^ *P*<0.1, *P<0.05, **P<0.01 and ***P<0.001.

Liver weight increased with HFD and was significantly lower in OPN-KO mice (Fig. 4A). Animals under HFD showed an altered cell structure, characterized by the presence of macrovesicular steatosis, whereas this effect was observed to a lesser extent in OPN-KO mice (Fig. 4B). Analysis of intrahepatic TG content showed elevated TG levels in WT mice with HFD and that OPN-deficiency prevented this increase (Fig. 4C). Moreover, HFD resulted in increased serum levels of glycerol and cholesterol, which were significantly reduced in mice lacking OPN (Table 1).

Lack of OPN was associated with a decrease in mRNA levels of the lipogenic transcription factors *Pparg* and *Srebf1*, their downstream target genes involved in the synthesis of FFA (*Fasn*), and TG (*Mogat1* and *Dgat2*), the formation of lipid droplets (*Cidec*) as well as in the VLDL uptake (*Vldlr*) (Fig. 4D–F). OPN-KO mice also reduced HFD-induced increase in AQP7 protein, an aquaporin involved in glycerol transport [26]. On the other hand, protein levels of UCP2 and UCP3, involved in fatty acid fuelling for energy expenditure, were increased with the HFD and with UCP3 being further increased in OPN-KO mice (Fig. 4G). The differential expression of other genes involved in lipid accumulation (*Anxa2, Cd36, Egfr*) caused by the HFD, were prevented by OPN-deficiency (Fig. 4D and Table S4). OPN-deficiency prevents the accumulation of intrahepatic TG and reduces the expression of molecules involved in the onset of liver steatosis.

### OPN-deletion decreases HFD-induced liver inflammation and fibrosis

**Figure 5:**
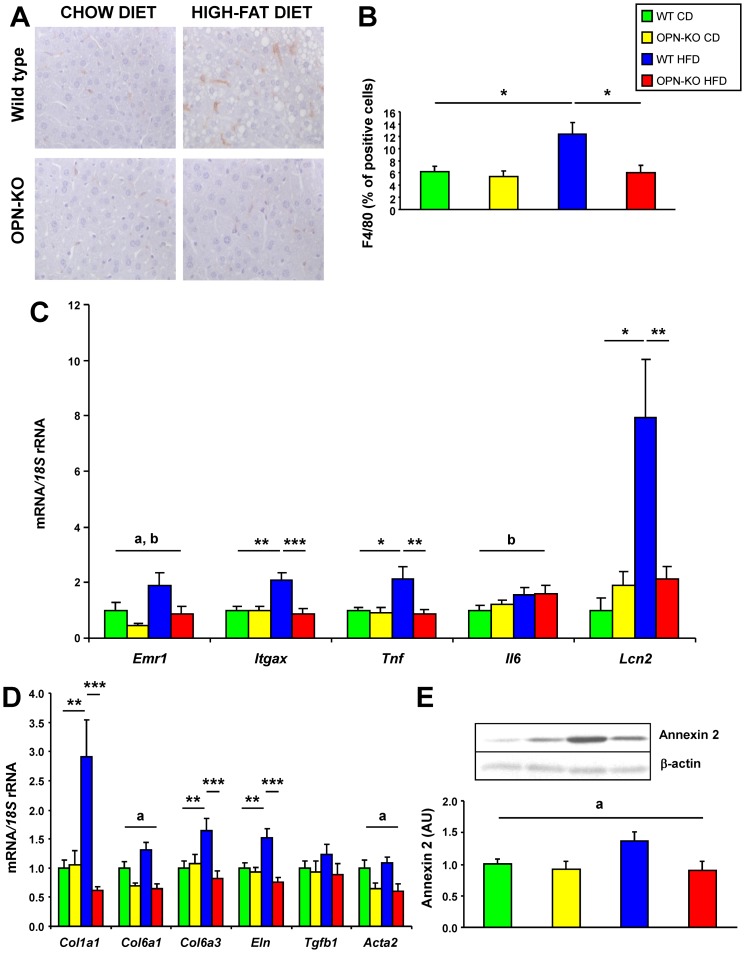
OPN-deficiency decreases HFD-induced liver inflammation and fibrosis. (A and B) Representative immunohistochemical staining of liver against the specific macrophage marker F4/80. Magnification 400X. Mean ± SEM of 5 animals. (C) Gene expression levels of *Emr1*, *Itgax*, *Tnf*, *Il6*, and *Lcn2* in liver after 20 weeks under CD or HFD. (D) Expression of *Col1a1*, *Col6a1*, *Col6a3, Eln*, *Tgfb1* and *Acta2* genes involved in fibrosis and (E) Annexin 2 protein. Mean ± SEM of 8–10 animals. Statistical differences were determined by two-way ANOVA, ^a^ *P*<0.05, effect of OPN deficiency; ^b^ *P*<0.05 effect of diet. If an interaction was detected one-way ANOVA followed by Tukey's HSD test was performed, *P<0.05, **P<0.01 and ***P<0.001.

Similar to the changes observed in adipose tissue, the macrophage number as well as F4/80 and CD11c mRNA in the liver were increased by HFD, while OPN deficiency prevented this increase (Fig. 5A–C). Analogously, *Tnf* mRNA increased with the HFD, being normal in OPN-KO mice. Lipocalin 2 (*Lcn2*) mRNA was upregulated with HFD, which was not observed in OPN-deficient mice (Fig. 5C). Mice lacking OPN have reduced hepatic macrophage infiltration, and *Tnf* and *Lcn2* expression compared to WT mice when fed a HFD. In the liver, an increase in size or number of fibrotic streak was not evident (data not shown). However, *Col1a1*, *Col6a3* and *Eln* mRNA increased with HFD, being normal in OPN-KO mice (Fig. 4D and 5D). α-SMA (*Acta2*) mRNA and annexin 2 mRNA and protein decreased in OPN-KO mice (Fig. 4D and 5D–E).

### Lack of OPN improves BAT function

**Figure 6:**
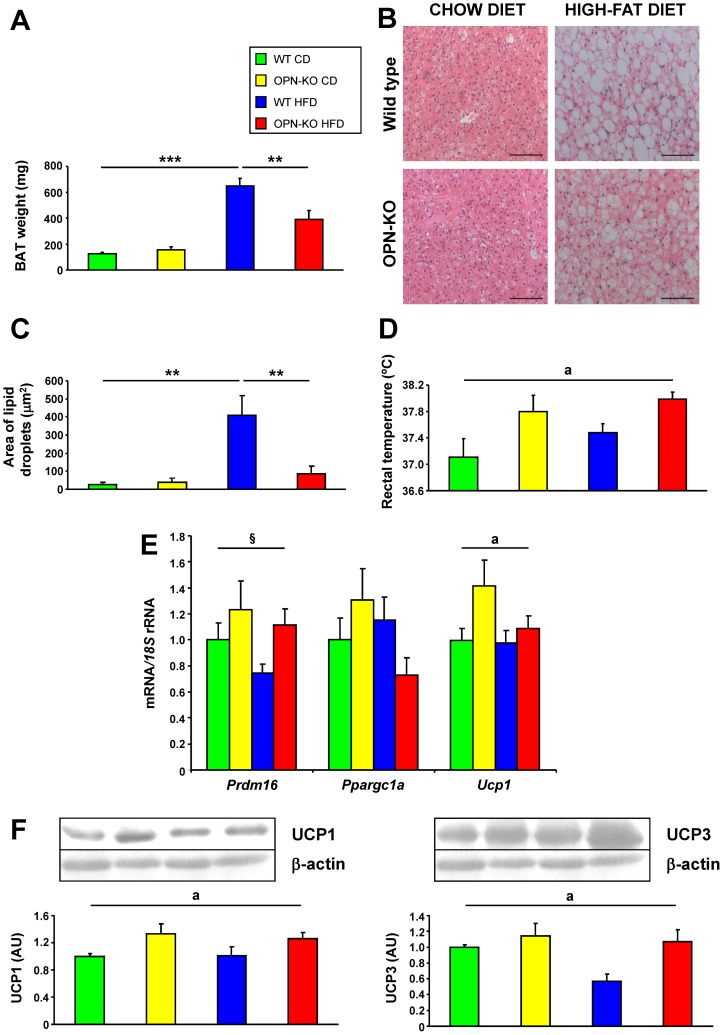
OPN-deficiency improves the structure and function of BAT. (A) BAT weight of the mice. Media ± SEM of 8–10 animals. (B) Representative images of histological sections of BAT from mice of different groups. The sections were stained with H–E. Magnification 200X. Scale bar, 100 µm. (C) Area of the lipid droplets in BAT. Mean ± SEM of 5 animals. (D) Rectal temperature. Mean ± SEM of 8–10 animals. (E) Expression of genes involved in thermogenesis, *Prdm16*, *Pgc1a* and *Ucp1* mRNA and (F) protein expression levels of UCP1 and UCP3 in BAT after 20 weeks of exposure to the chow diet or HFD. Mean ± SEM of 8–10 animals. Statistical differences were determined by two-way ANOVA, ^a^ *P*<0.05, effect of OPN deficiency. ^§^ *P* = 0.051, effect of OPN deficiency. If an interaction was detected one-way ANOVA followed by Tukey's HSD test was performed, **P<0.01 and ***P<0.001.

We next examined whether BAT function may explain the protection against HFD-induced obesity observed in OPN-KO mice. BAT weight increased by HFD, while OPN-deficiency partially prevented this increase (Fig. 6A). WT mice under HFD showed an altered cellular structure of BAT, characterized by the presence of large lipid droplets, increased number of unilocular fat cells and lower number of multilocular adipocytes (Fig. 6B–C). This effect was observed to a lesser extent in animals lacking OPN. Furthermore OPN-KO mice had a higher body temperature than their wild genotype counterparts (Fig. 6D). PRDM16, PGC1α and UCP1 are proteins involved in BAT adipocyte differentiation and thermogenesis regulation. *Prdm16* mRNA tended to increase (*P* = 0.051) in OPN-KO mice. Neither diet nor genotype affected *Ppargc1a* mRNA. *Ucp1* mRNA as well as UCP1 and UCP3 protein were significantly increased by the deficiency in OPN (Fig. 6E–F). OPN-KO mice with HFD have a better structure of BAT and an increase in body temperature and thermogenic proteins compared to WT mice.

## Discussion

In this study we provide evidence that OPN plays a major role in the adipose tissue expansion and liver steatosis that take place in HFD-induced obesity in mice. Furthermore, lack of OPN provides protection against inflammation, oxidative stress and fibrosis in both organs.

Circulating OPN levels of WT mice were not changed by HFD, which is in agreement with other reports [9], [12]. Nonetheless, this fact contrasts with data reported in obese patients [6], [9], [10] and with findings observed in mice by some groups [8]. However, we found that *Spp1* mRNA expression was dramatically increased in EWAT and liver of WT mice exposed to HFD, suggesting a more important pathophysiological role of OPN at the autocrine/paracrine level than systemically.

OPN binds to multiple integrin receptors and CD44, which is one of the main OPN receptors [2]. CD44 plays a causative role in the development of adipose tissue inflammation and insulin resistance in mice and has been related to type 2 diabetes in humans [27], [28]. In agreement with previously published results [28], *Cd44* increased with HFD, likely aggravating the effects triggered by OPN regarding inflammation and insulin resistance in EWAT and liver. We expected a compensatory increase of CD44 in OPN-KO mice, however it decreased to baseline levels likely because OPN promotes the expression of CD44 [29].

Lack of OPN blunted the HFD-induced increase in body weight and fat mass, despite a higher caloric intake, which agrees with previous reports [12] but contrasts with results reported by others [8], [30]. In this sense, OPN-KO mice showed higher total ghrelin levels and lower leptin concentrations, which could explain the higher food intake observed, since ghrelin and leptin show orexigenic and anorexigenic effects, respectively [31], [32]. Moreover, a reduction in adipocyte size in OPN-KO mice under the HFD was evidenced. We explored possible changes in main proteins involved in lipogenic or lipolytic pathways to explain the lower accumulation of adipose tissue in OPN-KO mice, but they remained unchanged. Therefore, changes in lipolytic or lipogenic pathways are unlikely explaining the observed effects on adipose tissue mass.

Adipose tissue remodeling is a continuous process that is pathologically accelerated in obesity [33]. MMP2 and MMP9 exert a pivotal role in adipose tissue remodeling that occurs during the development of obesity [34]. Previous studies from our group showed that gene expression of MMPs increases in obesity in parallel with a rise in OPN expression [21]. MMP2 and MMP9 activity increased with HFD, with this effect being more evident for MMP2, highlighting the importance of MMP2 in adipose tissue expansion. The diet-induced MMP activity increase occurred despite the reduction of MMP9 mRNA and the unchanged protein expression levels. The complex regulation of MMPs causes that levels of gene, protein and activity of MMPs, are not always concordant [35]–[37]. However, OPN-deletion prevented the increase of activity caused by HFD. It has been reported that OPN regulates gene and protein expression of MMP2 and MMP9 in neoplastic processes and cardiac remodeling [38]–[40]. Therefore, the decrease in adipose tissue remodeling via the reduction of MMPs activity may constitute a new mechanism by which OPN-deficiency protects against adipose tissue accretion caused by HFD.

Many studies have shown that obesity is associated with increased oxidative stress [41]. Moreover, OPN has been related with oxidative stress in mice and humans [42], [43]. NADPH oxidase is an enzyme that produces reactive oxygen species which is upregulated by HFD [41]. We observed that expression of *Nox1* and *Cybb* mRNA, and NOX2 protein levels were increased by the HFD, while OPN-deficiency protected against this increase. Moreover, lack of OPN prevented the increase of serum lipid peroxidation levels caused by HFD, suggesting that OPN-deficiency protects against systemic oxidative stress. Similar effects have been reported to take place in the kidney of OPN-KO mice, which are protected against aldosterone-induced oxidative stress [43]. Our data evidence a novel mechanism by which OPN-deletion exerts protective effects against the development of obesity-associated oxidative stress by decreasing lipid peroxidation and NADPH component levels.

The adipose tissue expansion that takes place in obesity is associated with macrophage accumulation [10]. OPN represents a potent chemoattractant and activator for macrophages [44]. Our data show that lack of OPN partly prevented the increase of macrophages, CLS and *Tnf* expression caused by HFD in EWAT, extending previously reported data [8], [45]. The decrease of *Cd11c* in OPN-KO mice with HFD showed that absence of OPN prevents the obesity-induced polarization switch of macrophages to a M1 proinflammatory state in EWAT. These findings are consistent with previous observations, reporting that the deletion of CD11c causes a decrease in CLS, improving insulin sensitivity through a decrease in inflammatory markers such as TNF-α and IL6 [23]. Taken together, our data evidence a lower macrophage inflammation, reduced phenotypic switch from M2 to M1 macrophages and decreased expression of proinflammatory cytokines in the adipose tissue of OPN-KO fed a HFD.

OPN has been related to fibrosis in different tissues such as the liver, heart, kidney and muscle [43], [46], [47]. Moreover, obesity has been related to the increased expression of collagens and the profibrotic cytokine TGF-β in adipose tissue, which has been associated with increased fibrosis [48], [49]. OPN-KO mice showed a reduction in HFD-induced fibrotic streaks as well as a decreased expression of collagens and *Tgfb1* in EWAT, showing for the first time that OPN-deficiency prevents the fibrosis induced by HFD in adipose tissue. No fibrotic structures were observed in the liver, probably due to the fact that fibrosis was still in its initial stages. In this sense, OPN-deficiency prevented the HFD-induced increase in extracellular matrix proteins such as *Col1a1*, *Col6a3* and *Eln*. Moreover *Col6a1* and markers of liver fibrosis such as α-SMA and annexin 2 [50] decreased by OPN-deletion regardless of diet. Our findings suggest the OPN-deficiency may prevent liver fibrosis, which is consistent with the observations reported by Syn and colleagues showing that OPN drives to fibrogenesis in NASH [47]. The reduced degree of inflammation observed in EWAT and liver of OPN-KO mice might be contributing to the lower fibrosis, since inflammation has been reported to be involved in the development of fibrosis in those organs [51], [52].

The absence of OPN reversed HFD-induced fatty liver, as shown by the reduction of lipogenic gene expression of *Srebf1, Mogat1* and *Dgat2* and TG accumulation in the liver. *Fasn* expression decreased in OPN-KO mice reflecting a reduced synthesis of FFA. OPN-deficiency prevented the increase of *Vldlr*, *Cidec* and *Pparg* caused by high-fat feeding, thus reflecting a defense against lipid accumulation. These data are consistent with those reported by Duval et al [53], showing that *Mogat1, Vldlr and Cidec* are increased in liver of mice with a high degree of hepatic steatosis. Reduced expression of *Cidec*, a protein involved in the formation of lipid droplets, has also been shown to be related to the protection against hepatic lipid accumulation in CD44-KO mice [28].OPN has been previously involved in the development of fatty liver and steatohepatitis in mice [5] and humans [54], in parallel with an increase in lipogenic genes. In addition, UCP3 protein increased with OPN-deletion, which is associated with a higher rate of lipid catabolism. Furthermore, the membrane protein AQP7, which correlates with hepatic steatosis [55], was also decreased in OPN-KO mice. Accordingly, OPN-deficiency improve shepatic steatosis induced by HFD.

Lack of OPN completely reversed the hepatic macrophage recruitment caused by HFD. The absence of OPN prevented the increase of *Cd11c* and *Tnf* mRNA showing that OPN-deficiency protects against obesity-induced liver inflammation. LCN2 is an early biomarker of liver damage and inflammation [56] related to obesity [21]. Moreover, *Lcn2*-KO mice exhibit improved insulin sensitivity [57]. Therefore, the decrease of *Lcn2* mRNA in OPN-KO mice may contribute to the reduced liver damage and inflammation as well as to the higher insulin sensitivity in the liver of these mice. The lower concentration of macrophages, together with the decrease of *Tnf* and *Lcn2* mRNA show that OPN-KO mice exhibit a better hepatic inflammatory profile than WT mice fed the HFD, similar to that observed in adipose tissue.

OPN-deletion protects against insulin resistance caused by HFD, as evidenced by the improvement in insulin levels, HOMA and IPITT. The lack of changes in *Irs1*, *Irs2* and *Slc2a4* in skeletal muscle, suggest that changes in adipose and liver could have a more important role in the improvement in insulin sensitivity observed in OPN-KO mice [30].

The reduced adiposity despite the increased food intake led us to hypothesize that OPN-KO mice exhibit an increased thermogenesis. In this respect, OPN-KO mice have a higher body temperature than WT mice. In addition, absence of OPN improved the brown-like phenotype of BAT in animals fed a HFD, which are characterized by a “white-like” appearance of brown fat. Moreover, OPN-deficient mice showed increased UCP1 and UCP3, proton transporters from the mitochondrial respiratory chain that generate heat by non-shivering thermogenesis and contribute to lower lipid accumulation in BAT as well as a lower body weight [58], [59]. Therefore, the increased body temperature and the changes in BAT morphology and expression of BAT-specific genes, identify the improvement of BAT function as a potential new mechanism whereby OPN-deficiency improves energy homeostasis.

In conclusion, OPN-deletion prevents the increase in body weight and adipose tissue expansion, in addition to decreasing macrophage infiltration, inflammation, oxidative stress, fibrosis and insulin resistance. Therefore, our results suggest that OPN could be an attractive target for the treatment of obesity and associated pathologies.

## Supporting Information

Figure S1 **HFD increases the expression of** ***Opn*** **and** ***Cd44*** **in EWAT and liver of WT mice.** (A) Circulating levels of OPN in the experimental groups, (B) *Opn* and (C) *Cd44* mRNA in EWAT, (D) *Opn* and (E) *Cd44* mRNA in liver of mice fed a CD or a HFD for 20 weeks. Mean ± SEM of 8–10 animals. Statistical differences were determined by Student's t test or two-way ANOVA as appropriate. If an interaction in the two-way ANOVA was detected, one-way ANOVA followed by Tukey's HSD test was performed. *P<0.05 and ***P<0.001.
(TIF)

Figure S2 **The expression of proteins involved in lipogenesis and lipolysis is not modified by OPN-deletion.** Protein kinase B (AKT1), 5′ AMP-activated protein kinase (AMPK), acetyl-coA carboxylase (ACC) and fatty acid synthase (FAS), involved in lipogenesis, and adipose triglyceride lipase (ATGL) and hormone-sensitive lipase (HSL), involved in lipolysis were analyzed in order to explore whether the changes observed in adipose mass were due to alterations in either lipolysis or lipogenesis. (A) Active AKT1 (ratio AKT1-P/AKT1), (B) active AMPK (ratio AMPK-P/AMPK), (C) active ACC (ratio ACC/ACC-P), (D) total amount of ATGL protein, (E) total amount of FAS protein, (F) active HSL (ratio HSL/HSL-P) and (G) *Pparg* mRNA in EWAT after 20 weeks under the CD or HFD. Mean ± SEM of 8-10 animals. Statistical differences were determined by two-way ANOVA, ^b^ *P*<0.05 effect of diet.
(TIF)

Figure S3 **Lack of OPN improves insulin sensitivity in mice fed a HFD.** (A) Serum glucose during intraperitoneal glucose tolerance test (IPGTT) and area under the curve (AUC) of the IPGTT, (B) serum glucose during intraperitoneal insulin tolerance test (IPITT) and AUC of the IPITT in animals of different experimental groups. Mean ± SEM of 5-6 animals. Statistical differences were determined by two-way ANOVA. ^b^ *P*<0.05 effect of diet. If an interaction was detected, one-way ANOVA followed by Tukey's HSD test was performed. *P<0.05. ¶*P*<0.05 WT CD vs WT HFD; ^#^ *P*<0.05 WT HFD vs OPN HFD. Gene expression levels of (C) *Irs1*, (D) *Irs2*, (E) *Slc2a4* and (F) *Ucp3* in gastrocnemius muscle of mice after 20 weeks of exposure to a CD or HFD. Mean ± SEM of 8-10 animals. Data were analyzed by two-way ANOVA, ^b^ *P*<0.05 effect of diet.
(TIF)

Figure S4 **Functional annotation network from IPA (** ***Ingenuity Pathway Analysis*** **) reveals an important role of MMPs and collagens in OPN's effect on HFD-induced adipose tissue expansion.** Colored genes are differentially expressed by OPN deletion in mice exposed to HFD. Green stands for those genes decreased with the lack of OPN while red reflects those genes increased with OPN deletion.
(TIF)

Table S1 **Sequences of the primers and probes used in the Real-Time PCR experiments.**
(PDF)

Table S2 **Sequences of the primers and probes used in the Real-Time PCR experiments.**
(PDF)

Table S3 **Selected genes differentially expressed in EWAT.**
(PDF)

Table S4 **Selected genes differentially expressed in the liver.**
(PDF)
