# Supplementary figures and images for: Osteopontin Deletion Prevents the Development of Obesity and Hepatic Steatosis via Impaired Adipose Tissue Matrix Remodeling and Reduced Inflammation and Fibrosis in Adipose Tissue and Liver in Mice
Source: PLoS One. 2014 May 28;9(5):e98398. doi: 10.1371/journal.pone.0098398 (PMC4037189; doi:10.1371/journal.pone.0098398)

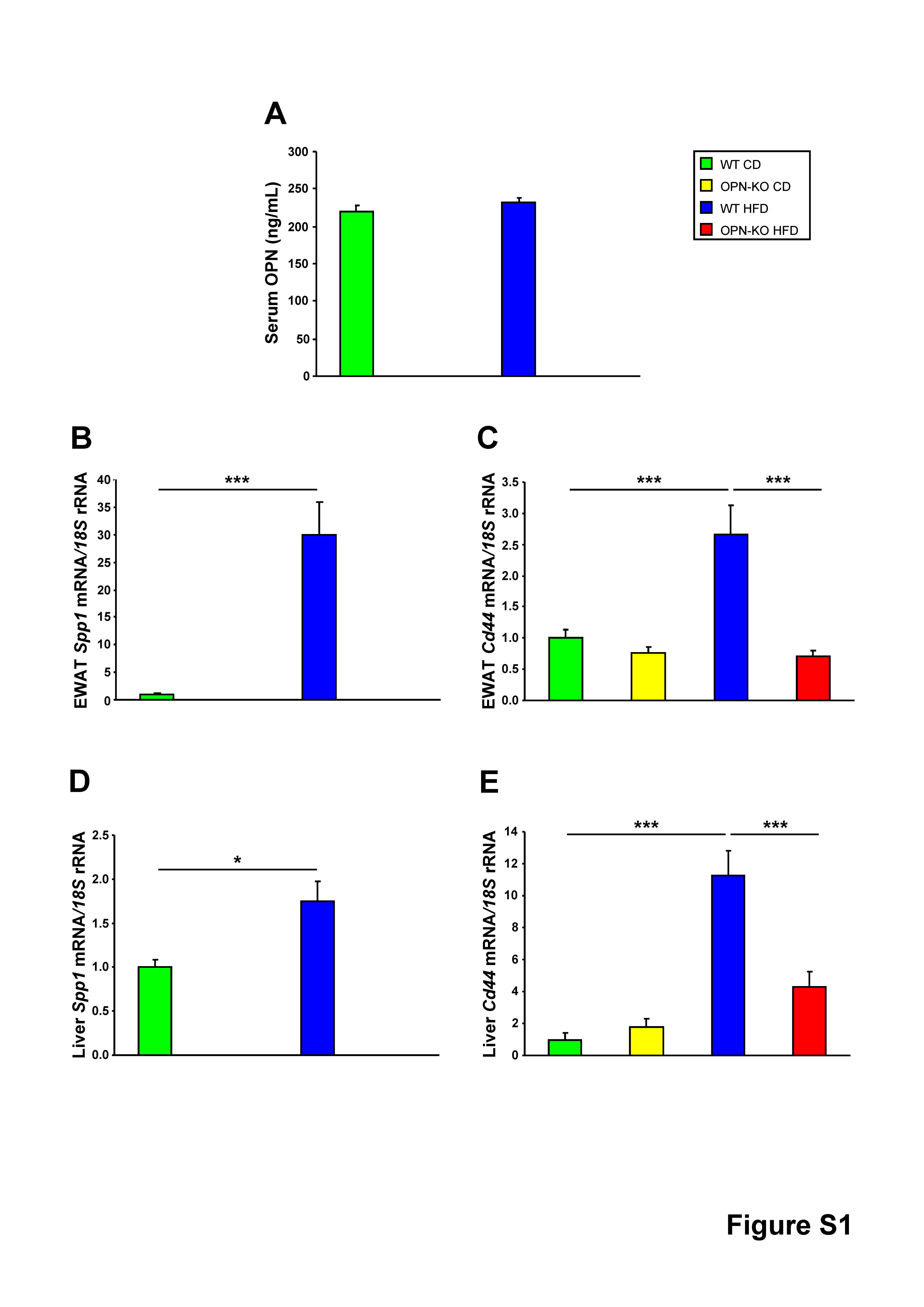

Supplement: Figure S1 — HFD increases the expression of Opn and Cd44 in EWAT and liver of WT mice. (A) Circulating levels of OPN in the experimental groups, (B) Opn and (C) Cd44 mRNA in EWAT, (D) Opn and (E) Cd44 mRNA in liver of mice fed a CD or a HFD for 20 weeks. Mean ± SEM of 8–10 animals. Statistical differences were determined by Student's t test or two-way ANOVA as appropriate. If an interaction in the two-way ANOVA was detected, one-way ANOVA followed by Tukey's HSD test was performed. *P<0.05 and ***P<0.001. (TIF) [file pone.0098398.s001.tif]

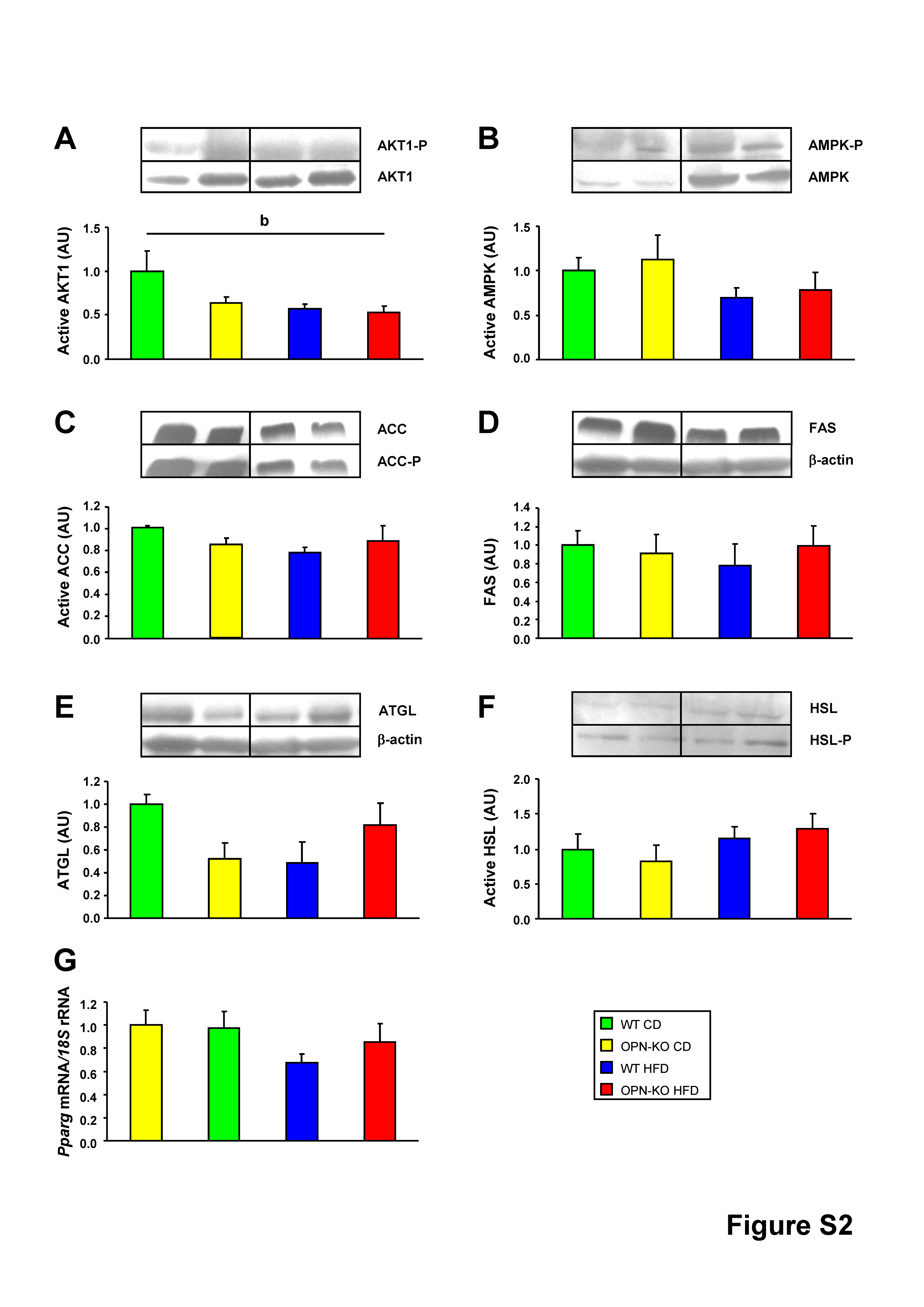

Supplement: Figure S2 — The expression of proteins involved in lipogenesis and lipolysis is not modified by OPN-deletion. Protein kinase B (AKT1), 5′ AMP-activated protein kinase (AMPK), acetyl-coA carboxylase (ACC) and fatty acid synthase (FAS), involved in lipogenesis, and adipose triglyceride lipase (ATGL) and hormone-sensitive lipase (HSL), involved in lipolysis were analyzed in order to explore whether the changes observed in adipose mass were due to alterations in either lipolysis or lipogenesis. (A) Active AKT1 (ratio AKT1-P/AKT1), (B) active AMPK (ratio AMPK-P/AMPK), (C) active ACC (ratio ACC/ACC-P), (D) total amount of ATGL protein, (E) total amount of FAS protein, (F) active HSL (ratio HSL/HSL-P) and (G) Pparg mRNA in EWAT after 20 weeks under the CD or HFD. Mean ± SEM of 8-10 animals. Statistical differences were determined by two-way ANOVA, b P<0.05 effect of diet. (TIF) [file pone.0098398.s002.tif]

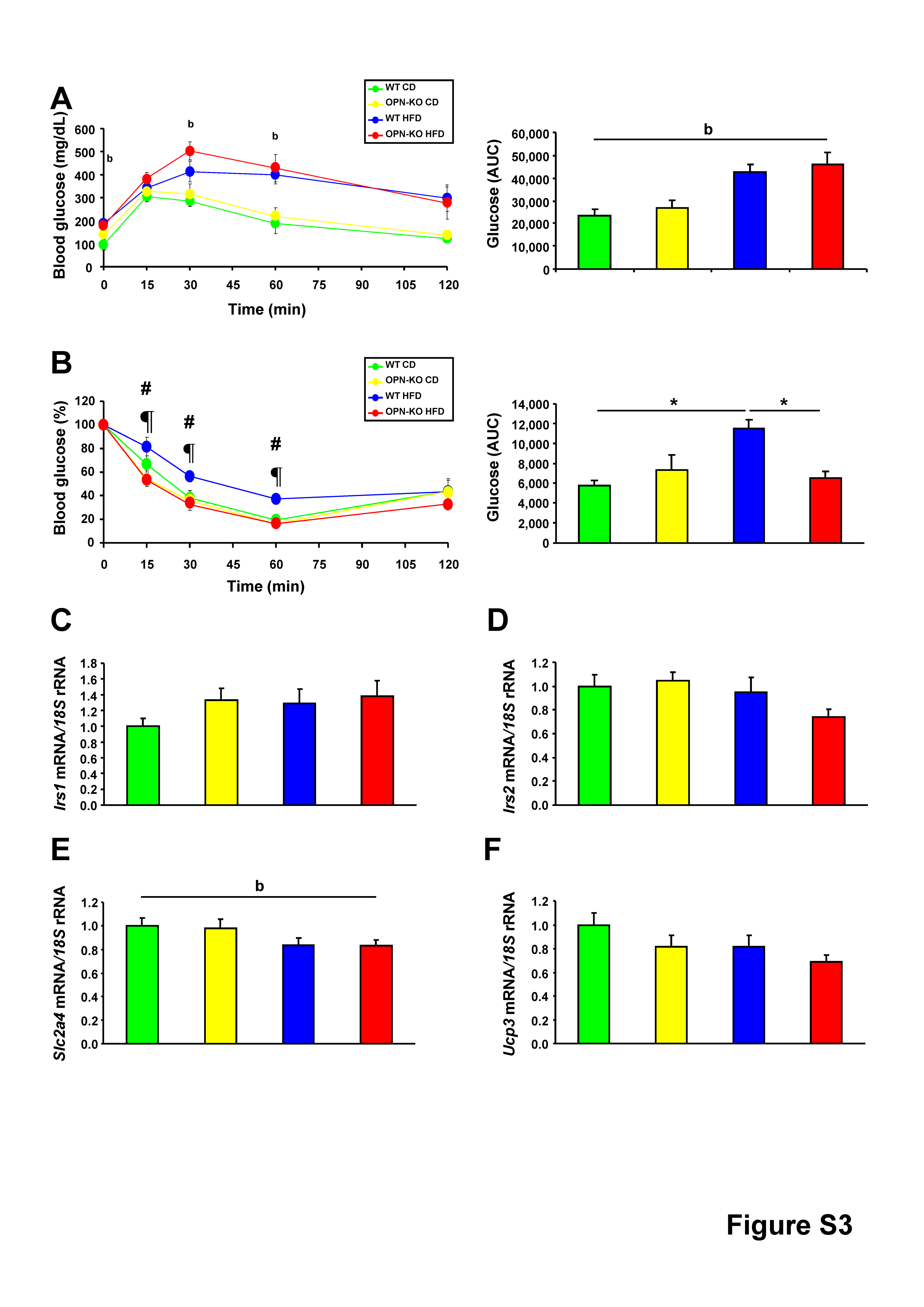

Supplement: Figure S3 — Lack of OPN improves insulin sensitivity in mice fed a HFD. (A) Serum glucose during intraperitoneal glucose tolerance test (IPGTT) and area under the curve (AUC) of the IPGTT, (B) serum glucose during intraperitoneal insulin tolerance test (IPITT) and AUC of the IPITT in animals of different experimental groups. Mean ± SEM of 5-6 animals. Statistical differences were determined by two-way ANOVA. b P<0.05 effect of diet. If an interaction was detected, one-way ANOVA followed by Tukey's HSD test was performed. *P<0.05. ¶P<0.05 WT CD vs WT HFD; # P<0.05 WT HFD vs OPN HFD. Gene expression levels of (C) Irs1, (D) Irs2, (E) Slc2a4 and (F) Ucp3 in gastrocnemius muscle of mice after 20 weeks of exposure to a CD or HFD. Mean ± SEM of 8-10 animals. Data were analyzed by two-way ANOVA, b P<0.05 effect of diet. (TIF) [file pone.0098398.s003.tif]

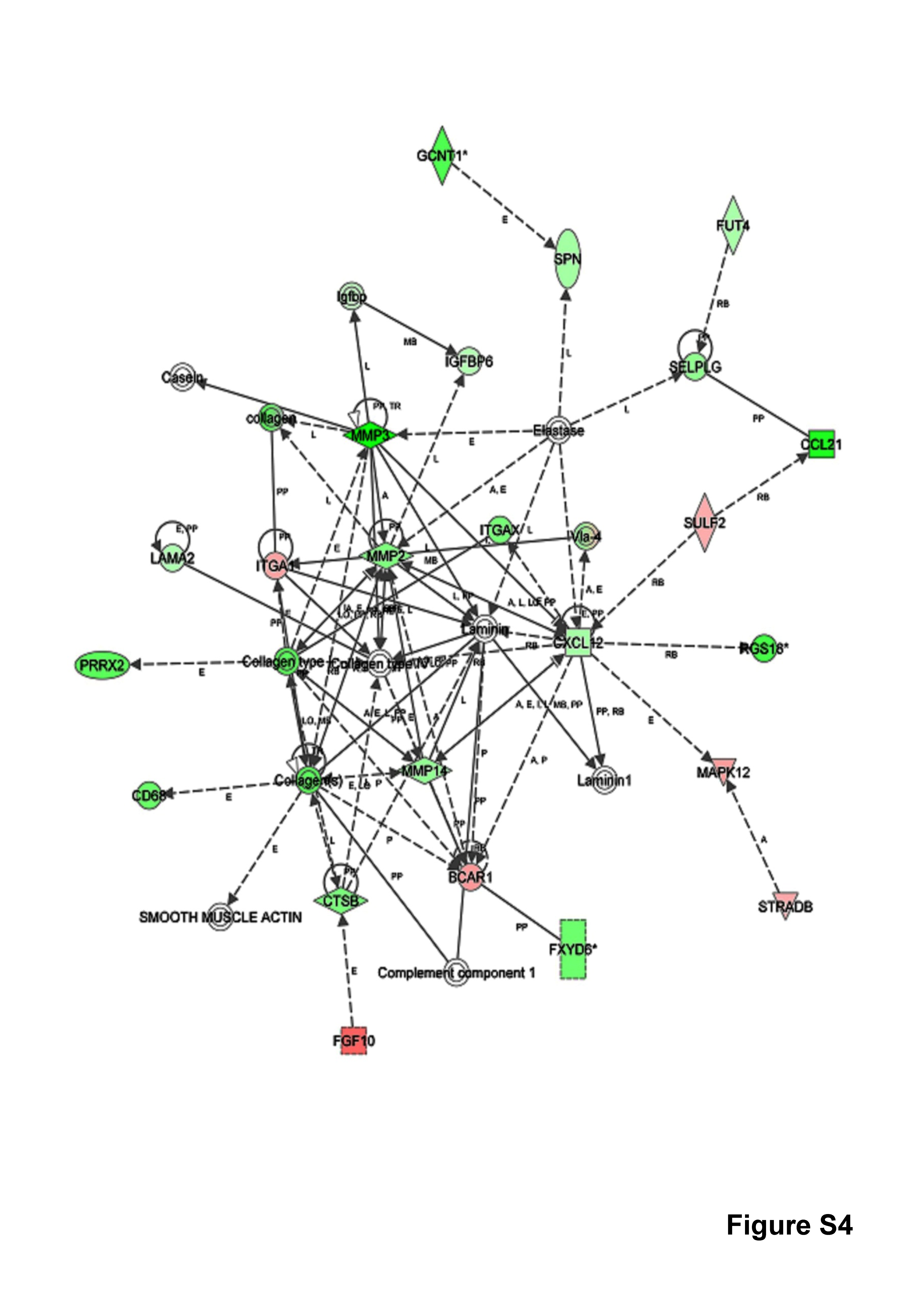

Supplement: Figure S4 — Functional annotation network from IPA ( Ingenuity Pathway Analysis ) reveals an important role of MMPs and collagens in OPN's effect on HFD-induced adipose tissue expansion. Colored genes are differentially expressed by OPN deletion in mice exposed to HFD. Green stands for those genes decreased with the lack of OPN while red reflects those genes increased with OPN deletion. (TIF) [file pone.0098398.s004.tif]
