# Supplementary material for: Osteopontin Deletion Prevents the Development of Obesity and Hepatic Steatosis via Impaired Adipose Tissue Matrix Remodeling and Reduced Inflammation and Fibrosis in Adipose Tissue and Liver in Mice
Source: PLoS One. 2014 May 28;9(5):e98398. doi: 10.1371/journal.pone.0098398 (PMC4037189; doi:10.1371/journal.pone.0098398)
Supplement: Table S1 — Sequences of the primers and probes used in the Real-Time PCR experiments. (PDF) [file pone.0098398.s005.pdf]

Table S1. Sequences of the Primers and Probes

Gene (GenBank accession)	Oligonucleotide sequence (5'-3')
***Spp1* (NM_009263)**	
Forward	TTTGCCGTTTGGCATTGC
Reverse	TGGGTGCAGGCTGTAAAGCT
TaqMan Probe	FAM-TCCTCCCTCCCGGTGAAAGT-TAMRA
***Cd44* (NM_009851)**	
Forward	AGAGCCGGAAGAAGACGAAAAC
Reverse	TCCACCTCTTCTTGCATCTTTAGC
TaqMan Probe	FAM-AGCTGATCTGGTTCCCACT-TAMRA
***Irs1* (NM_010570)**	
Forward	GATGATGTCACCCAGTGGTAGTTG
Reverse	TCCCATAGCTGCTCCCAGAA
TaqMan Probe	FAM-AGCAGCAGTAGCAGCATCAGCGCA-TAMRA
***Irs2* (NM_001081212)**	
Forward	TAAACGGAGGTGGCTACAAAGC
Reverse	GCTTAGGGTCTGGGTTCTCCAT
TaqMan Probe	FAM-CATGCGAATGTGGTGTGGCTCCAA-TAMRA
***Slc2a4* (NM_009204)**	
Forward	GTCCTGAGAGCCCCAGATAC
Reverse	TCCAACTTCCGTTTCTCATCCT
TaqMan Probe	FAM-CCTGCCCGAAAGAGTCTAAAGCGCC-TAMRA
***Ucp3* (NM_009464)**	
Forward	GACCTACGACATCATCAAGGAGAAGT
Reverse	CTCCAAAGGCAGAGACAAAGTGA
TaqMan Probe	FAM-TCTCACCTGTTTACTGACAACTTCCC-TAMRA
***Mmp2* (NM_008610)**	
Forward	TGCTCCACCACATACAACTTTGA
Reverse	GAAGCGGAACGGGAACTTG
TaqMan Probe	FAM-TCTGCCCCCATGAAGCCTTGTTTACC-TAMRA
***Mmp9* (NM_013599)**	
Forward	CCAAAGACCTGAAAACCTCCAA
Reverse	GCCCGGGTGTAACCATAGC
TaqMan Probe	FAM-CACCCAGCTGGCAGAGGCATACTTGT-TAMRA
***Emr1* (NM_010130)**	
Forward	CAAGATTCTCTTCCTCACCGGTAT
Reverse	GCAGGCGAGGAAAAGATAGTGTAG
TaqMan Probe	FAM-CAACCAGACGGCTTGTGCCATCATT-TAMRA
***Itgax* (NM_021334)**	
Forward	CTGGACTTTGTTAAAGCTGTGATGAG
Reverse	GACGTGGAGATGAAGTTGTTGAAA
TaqMan Probe	CCTAGCACACGGTTCTCCCTGATGCA-TAMRA
***Tnf* (NM_013693)**	
Forward	CCAGACCCTCACACTCAGATCAT
Reverse	ACTCCAGCTGCTCCTCCACTT
TaqMan Probe	FAM-CCTGTAGCCCACGTCGTAGCAAACCA-TAMRA
***Il6* (NM_031168)**	
Forward	CGGAGGCTTAATTACACATGTTCTC
Reverse	CAGTTTGGTAGCATCCATCATTTCT

TaqMan Probe	FAM-CGTGGAAATGAGAAAAGAGTTGTGCAATGG-TAMRA
***Adipoq* (NM_009605)**	
Forward	AAGGAGATGCAGGTCTTCTTGGT
Reverse	CACTGAACGCTGAGCGATACAT
TaqMan Probe	FAM-TGGAATGACAGGAGCTGAAGGGCCA-TAMRA
***Nox1* (NM_172203)**	
Forward	TTATCGCTCCCAGCAGAAGGT
Reverse	CATGCTAAAGCCTCGCTTCCT
TaqMan Probe	FAM-ATTACCAAGGTTGTCATGCACCCA-TAMRA
***Cybb* (NM_007807)**	
Forward	TGTGTCGAAATCTGCTCTCCTTT
Reverse	AAAGTGAGGTTCCTGTCCAGTTGT
TaqMan Probe	FAM-AGTGCGTGTTGCTCGACAAGGAT-TAMRA
***Col1a1* (NM_007742)**	
Forward	TGTCCCAACCCCCAAAGAC
Reverse	GGTCCCTCGACTCCTACATCTTC
TaqMan Probe	FAM-CTGCCCGGAAGAATACGTATCACCAAACTC-TAMRA
***Col6a1* (NM_009933)**	
Forward	CACCTGGGCCAGATGAGTGT
Reverse	CCAGCACGAAGAGGATGTCAA
TaqMan Probe	FAM-AAATGTGCTCCTGCTGTGA-TAMRA
***Col6a3* (NM_001243008)**	
Forward	TGATGGCACCTCTCAGGACTCT
Reverse	TTGTCGGAGCCATCCAAAAG
TaqMan Probe	FAM-CCACGGAAGTTCACGTAA-TAMRA
***Tgfb1* (NM_011480)**	
Forward	TCCCAAGAGCCCTGCACTT
Reverse	GTCCACAAAGAAACGGTGACCTA
TaqMan Probe	FAM-TTGACACGTTTCTTCCTGAGCAGCGC-TAMRA
***Eln* (NM_007925)**	
Forward	CAAGACCTGGCTTTGGACTTTCT
Reverse	CAAAGCAGCCCCCACCTT
TaqMan Probe	FAM-CCATTTATCCAGGTGGTGGT-TAMRA
***Acta2* (NM_007392)**	
Forward	GTATCCGATAGAACACGGCATCA
Reverse	GGCCACACGAAGCTCGTTATAG
TaqMan Probe	FAM-CATGGAAAAGATCTGGCACC-TAMRA
***Pparg* (NM_001127330)**	
Forward	GCTTCCACTATGGAGTTCATGCTT
Reverse	ATCCGGCAGTTAAGATCACACCTA
TaqMan Probe	AGGATGCAAGGGTTTTTTCCGA-TAMRA
***Srebf1* (NM_011480)**	
Forward	TCCCAAGAGCCCTGCACTT
Reverse	GTCCACAAAGAAACGGTGACCTA
TaqMan Probe	FAM-TTGACACGTTTCTTCCTGAGCAGCGC-TAMRA
***Fasn* (NM_007988)**	
Forward	GATGACATCGTGCATGCCTTT
Reverse	GTCAGGTTTCAGTCCCACAGAAGT
TaqMan Probe	FAM-CTGCCATCCAGATTGCCCTCATCG-TAMRA
***Mogat1* (NM_026713)**	
Forward	GTTTCCCGTTGTTCCGAGAATAT

Reverse	TGCTCAGCACATGAGACAAACTC
TaqMan Probe	FAM-TGATGAGTAACGGGCCGGTTTCAGTG-TAMRA
Dgat2 **(NM_026384)**	
Forward	GAAGAACCGCAAAGGCTTTGT
Reverse	GATCACCTGCTTGTATACCTCATTCTC
TaqMan Probe	FAM-AGCTGATCTGGTTCCCACT-TAMRA
Cidec **(NM_178373)**	
Forward	CCTGGCAAAAGATACCATGTTCA
Reverse	GCTTCTGGGAAAGGGCTAGCT
TaqMan Probe	FAM-CCCCATCAGAACAGCGCAAGAAGAGAG-TAMRA
Vldlr **(NM_001161420)**	
Forward	TCGTGGCTATCAAATGGATCTTG
Reverse	GGCCAATCTTCCTGATGTCTCTT
TaqMan Probe	CGTGTGCAAGGCAGTAGGCAAAGAGC-TAMRA
Lcn2 **(NM_008491)**	
Forward	TTGATCCCTGCCCCATCTC
Reverse	CTGTTTTTTTCTGGACCGCATT
TaqMan Probe	FAM-TCCGGAGCGATCAGTTCCGGG-TAMRA
Prdm16 **(NM_027504)**	
Forward	GATGGGAGATGCTGACGGATAC
Reverse	CTCGCTACCCAAGTCTTCAGACAT
TaqMan Probe	FAM-CATCCCAGGAGAGCTGCATCAAAAAGC-TAMRA
Ppargc1a **(NM_008904)**	
Forward	GTCTGAAAGGGCCAAACAGAG
Reverse	TCAATTCTGTCCGCGTTGTG
TaqMan Probe	FAM-AGCAGAAAGCAATTGAAGAGCGCCGT-TAMRA
Ucp1 **(NM_009463)**	
Forward	CGATGTCCATGTACACCAAGGA
Reverse	ACCCGAGTCGCAGAAAAGAAG
TaqMan Probe	FAM-ACCGACGGCCTTTTTCAAAGGGTTTG-TAMRA

Spp1, secreted phosphoprotein 1; ***Cd44***, Cd44 antigen; ***Irs1***, insulin receptor substrate 1; ***Irs2***, insulin receptor substrate 2; ***Slc2a4***, solute carrier family 2, member 4 (Glut4); ***Ucp3***, uncoupling protein 3; ***Mmp2***, matrix metalloproteinase 2; ***Mmp9***, matrix metalloproteinase 9; ***Emr1***, EGF-like module containing, mucin-like, hormone receptor-like sequence 1; ***Itgax***, integrin alpha X (Cd11c); ***Tnf***, tumor necrosis factor alpha; ***Il6***, interleukin 6; ***Adipoq***, adiponectin; ***Nox1***, NADPH oxidase 1; ***Cybb***, cytochrome b-245, beta polypeptide (Nox2); ***Col1a1***, collagen, type I, alpha 1; ***Col6a1***, collagen, type VI, alpha 1; ***Col6a3***, collagen, type VI, alpha 3; **Tgfb1**, transforming growth factor, beta 1; **Eln**, elastin; **Acta2**, actin, alpha 2, smoth muscle, aorta (alpha-Sma); ***Pparg***, peroxisome proliferators-activated receptor gamma; ***Srebf1***, sterol regulatory element binding transcription factor 1; ***Fasn***, fatty acid synthase; ***Mogat1***, monoacylglycerol O-acyltransferase 1; ***Dgat2***, diacylglycerol O-acyltransferase 2; ***Cidec***, cell death-inducing DFFA-like effector c; ***Vldlr***, very low density lipoprotein receptor; ***Lcn2***, lipocalin 2; ***Prdm16***, PR domain containing 16; ***Ppargc1a***, peroxisome proliferators-activated receptor gamma coactivator 1 alpha; ***Ucp1***, uncoupling protein1.
