# Supplementary material for: Osteopontin Deletion Prevents the Development of Obesity and Hepatic Steatosis via Impaired Adipose Tissue Matrix Remodeling and Reduced Inflammation and Fibrosis in Adipose Tissue and Liver in Mice
Source: PLoS One. 2014 May 28;9(5):e98398. doi: 10.1371/journal.pone.0098398 (PMC4037189; doi:10.1371/journal.pone.0098398)
Supplement: Table S2 — Sequences of the primers and probes used in the Real-Time PCR experiments. (PDF) [file pone.0098398.s006.pdf]

Table S2. Organ Weights of Experimental Animals

	Chow diet		High-fat diet	
	**Wild type**	**OPN-KO**	**Wild type**	**OPN-KO**
Heart (mg) [a,b]	143 ± 6	184 ± 7	170 ± 5	190 ± 7
Spleen (mg) [a]	67 ± 5	115 ± 12	86 ± 6	106 ± 9
Kidney (mg) [a,b]	187 ± 8	222 ± 7	212 ± 10	224 ± 9
Adrenal glands (mg) [a]	1.5 ± 0.2	1.9 ± 0.2	1.5 ± 0.1	1.9 ± 0.2
Testicle (mg)	82 ± 6	90 ± 2	90 ± 4	96 ± 3
Brain (mg) [a]	447 ± 6	423 ± 8	438 ± 2	436 ± 5
EDL (mg)	11.6 ± 1.0	12.7 ± 0.6	11.7 ± 0.6	12.2 ± 0.8
Soleus (mg) [b]	9.5 ± 0.9	9.7 ± 0.4	11.9 ± 0.8	10.3 ± 0.6
Gastrocnemius (mg) [a]	161 ± 7	167 ± 5	166 ± 3	180 ± 4
Femur (mg) [a]	82 ± 3	86 ± 3	80 ± 2	89 ± 3
Tibia (mg) [a]	61 ± 2	65 ± 3	54 ± 1	66 ± 2
Body length (cm)	10.2 ± 0.1	10.4 ± 0.4	10.4 ± 0.1	10.7 ± 0.2
Femur length (mm) [a]	15.6 ± 0.6	16.8 ± 0.4	15.8 ± 0.3	17.0 ± 0.3
Tibia length (mm)	18.8 ± 0.5	19.4 ± 0.3	18.7 ± 0.2	18.9 ± 0.2

Mean ± SEM of 8-10 animals. Statistical differences were determined by two-way ANOVA.

[a] $P<0.05$, effect of OPN deletion; [b] $P<0.05$ effect of diet.
