# Supplementary material for: Osteopontin Deletion Prevents the Development of Obesity and Hepatic Steatosis via Impaired Adipose Tissue Matrix Remodeling and Reduced Inflammation and Fibrosis in Adipose Tissue and Liver in Mice
Source: PLoS One. 2014 May 28;9(5):e98398. doi: 10.1371/journal.pone.0098398 (PMC4037189; doi:10.1371/journal.pone.0098398)
Supplement: Table S3 — Selected genes differentially expressed in EWAT. (PDF) [file pone.0098398.s007.pdf]

Table S3. Selected Genes Differentially Expressed in EWAT						
**GenBank accesion number**	**Gene Symbol**	**Gene name**	**Wild type CD**	**OPN-KO CD**	**Wild type HFD**	**OPN-KO HFD**
NM_020258	*Slc37a2*	Solute carrier family 37 (glycerol-3-phosphate transporter), member 2	1.00	1.38	30.55	4.24
NM_007498	*Atf3*	Activating transcription factor 3	1.00	1.20	27.60	4.08
NM_001044384	*Timp1*	Tissue inhibitor of metalloproteinase 1, transcript variant 1	1.00	2.18	26.62	3.29
NM_010130	*Emr1*	EGF-like module containing, mucin-like, hormone receptor-like sequence 1	1.00	2.07	23.77	2.64
NM_001033245	*Hk3*	Hexokinase 3	1.00	1.26	18.88	3.27
NM_009853	*Cd68*	CD68 antigen	1.00	2.42	16.56	4.15
NM_030682	*Tlr1*	Toll-like receptor 1	1.00	1.48	14.26	3.00
NM_009230	*Soat1*	Sterol O-acyltransferase 1	1.00	1.89	13.46	2.88
NM_009914	*Ccr3*	Chemokine (C-C motif) receptor 3	1.00	2.94	13.22	1.23
NM_133212	*Tlr8*	Toll-like receptor 8	1.00	1.16	11.52	2.59
NM_010809	*Mmp3*	Matrix metallopeptidase 3	1.00	1.78	9.83	1.23
NM_009917	*Ccr5*	Chemokine (C-C motif) receptor 5	1.00	1.60	8.72	3.03
AK037554	*Itgax*	NOD-derived CD11c +ve dendritic cells cDNA, RIKEN full-length enriched library, clone:F630002M04 product:integrin alpha X, full insert sequence	1.00	1.58	7.47	2.20
NM_007742	*Col1a1*	Procollagen, type I, alpha 1	1.00	1.77	7.42	1.65
NM_007798	*Ctsb*	Cathepsin B	1.00	1.06	7.27	2.20
NM_008855	*Prkcb1*	Protein kinase C, beta 1	1.00	1.23	6.84	1.57
NM_021460	*Lip1*	Lysosomal acid lipase 1	1.00	1.13	5.78	1.78
NM_011604	*Tlr6*	Toll-like receptor 6	1.00	1.28	5.61	2.02
NM_133211	*Tlr7*	Toll-like receptor 7	1.00	1.44	5.24	1.35
NM_008610	*Mmp2*	Matrix metallopeptidase 2	1.00	1.77	4.24	1.36
NM_007739	*Col8a1*	Procollagen, type VIII, alpha 1	1.00	1.28	3.93	1.25
NM_015763	*Lpin1*	Lipin 1, transcript variant 2	1.00	0.74	0.28	0.69
NM_009204	*Slc2a4*	Solute carrier family 2 (facilitated glucose transporter), member 4	1.00	0.74	0.27	0.67
NM_178934	*Slc2a12*	Solute carrier family 2 (facilitated glucose transporter), member 12	1.00	0.85	0.24	0.87
NM_023184	*Klf15*	Kruppel-like factor 15	1.00	0.78	0.22	0.72

Genes sorted from largest to smallest value of fold change (wild type with HFD compared to wild type with CD)
