# Supplementary material for: Osteopontin Deletion Prevents the Development of Obesity and Hepatic Steatosis via Impaired Adipose Tissue Matrix Remodeling and Reduced Inflammation and Fibrosis in Adipose Tissue and Liver in Mice
Source: PLoS One. 2014 May 28;9(5):e98398. doi: 10.1371/journal.pone.0098398 (PMC4037189; doi:10.1371/journal.pone.0098398)
Supplement: Table S4 — Selected genes differentially expressed in the liver. (PDF) [file pone.0098398.s008.pdf]

Table S4. Selected Genes Differentially Expressed in the Liver						
GenBank accesion number	Gene Symbol	Gene name	Wild type CD	OPN-KO CD	Wild type HFD	OPN-KO HFD
NM_013703	*Vldlr*	Very low density lipoprotein receptor	1.00	0.67	9.57	2.12
NM_007585	*Anxa2*	Annexin A2	1.00	0.91	6.05	1.37
NM_008484	*Lamb3*	Laminin, beta 3	1.00	1.32	4.37	1.49
NM_007742	*Col1a1*	Procollagen, type I, alpha 1	1.00	1.54	2.66	0.98
NM_016704	*C6*	Complement component 6	1.00	0.88	0.29	0.89
NM_172498	*Ptk2b*	PTK2 protein tyrosine kinase 2 beta	1.00	0.96	0.23	0.91
NM_133882	*C8b*	Complement component 8, beta subunit	1.00	0.83	0.23	0.69
NM_007912	*Egfr*	Epidermal growth factor receptor	1.00	1.06	0.22	0.96
NM_008294	*Hsd3b4*	Hydroxy-delta-5-steroid dehydrogenase, 3 beta- and steroid delta-isomerase 4	1.00	0.77	0.02	1.32
NM_008295	*Hsd3b5*	Hydroxy-delta-5-steroid dehydrogenase, 3 beta- and steroid delta-isomerase [illegible]	1.00	0.74	0.01	1.29

Genes sorted from largest to smallest value of fold change (wild type with HFD compared to wild type with CD)
